# Comparative efficacy of acupuncture, venesection, and physical therapy on chronic low back pain outcomes: a randomized clinical trial

**DOI:** 10.1097/MS9.0000000000001944

**Published:** 2024-03-18

**Authors:** Moein Jamali Dastjerdi, Mohaddeseh Azadvari, Gholamreza Kordafshari, Bai-Xiao Zhao, Mohammad Sadegh Adel-Mehraban, Reihane Alipour, Mehrdad Karimi, Amir Hooman Kazemi, Arman Sourani, Ali Vafaie Sefti

**Affiliations:** a Department of Traditional Medicine, School of Persian medicine, Tehran University of Medical Sciences, Tehran, Iran; b Department of Physical Medicine and Rehabilitation, Imam Khomeini Hospital, Tehran University of Medical Sciences, Tehran, Iran; c School of Traditional Chinese Medicine, Beijing University of Chinese Medicine, Beijing, China; d Traditional Persian Medicine and Complementary Medicine (PerCoMed) Student Association, Students’ Scientific Research Center (SSRC), Tehran University of Medical Sciences, Tehran, Iran; e Department of Traditional Medicine, School of Persian medicine, Shahid Beheshti University of Medical Sciences, Tehran, Iran; f International School, Beijing University of Chinese Medicine, Beijing, China; g Department of Neurosurgery, School of Medicine, Isfahan University of Medical Sciences, Isfahan, Iran

**Keywords:** Acupuncture, chronic low back pain, oswestry disability index, physical therapy modalities, traditional chinese medicine, traditional persian medicine, venesection

## Abstract

**Objective:**

Chronic low back pain (CLBP) imposes considerable financial and social burden with poor response to medical and surgical treatments. Alternatively, acupuncture and venesection(Fasd) are traditionally used to alleviate nociceptive and musculoskeletal pains. This study aimed to evaluate the effectiveness and the safety of acupuncture and venesection on CLBP and patient functionality.

**Methods:**

The current study was a single-blinded, randomized clinical trial with balanced allocation, conducted in the Department of Physical Medicine & Rehabilitation Medicine, in 2022. One hundred five CLBP patients who had no back pain-attributable structural or major diseases were randomly allocated into three parallel arms and received either physical therapy (PTG), acupuncture (APG), or venesection (VSG). Pain severity and functional aspects were evaluated using the visual analogue scale (VAS) and Oswestry disability index (ODI) during the study. VAS and ODI scores were defined as the primary outcomes.

**Results:**

Ninety-five patients were reviewed in the final analysis (PTG=33, APG=30, VSG=31). Demographic data showed equal group distribution. Statistical analysis showed all procedures had reduced VAS score immediately after the first session, after the last session, and after follow-up; however, APG and VSG values were significantly lower (*P*<0.05). Pain reduction results in follow-up period were more sustainable in APG and VSG as compared to PTG (*P*<0.01). ODI results revealed global improvement after the last session of the treatment in all groups, while APG had more significant results (*P*<0.05). During the follow-up period, ODI still tended to decrease in VSG, non-significantly increased in APG, and significantly increased in PTG. Only two patients reported fainting after receiving venesection.

**Conclusion:**

Considering the pain and functional scores, both acupuncture and venesection can reproduce reliable results. Acupuncture and venesection both have sustained effects on pain and daily function of the patients even after treatment termination, while physical therapy had more relapse in pain and functional limitations.

## Background

Highlights

- Acupuncture and venesection decreased pain severity (visual analogue scale) in low back pain.
- Acupuncture and venesection declined oswestry low back pain disability index.
- These methods were more effective than physical therapy.
- The lowest relapse rate was in the venesection group.

Low back pain (LBP), is one of the leading causes of progressive physical and psychological problems among adults with musculoskeletal disorders^1,2^. Although most of the patients recover within days, about 30% of LBPs, persist for more than 3 months and become chronic LBP (CLBP)^1^. LBP imposes a significant financial and social burden, with an estimated annual cost of $200 billion in the United States^2^. Although non-steroidal anti-inflammatory drugs (NSAIDs) may be helpful in short-term use, there is a growing concern about their potential adverse side effects^3^. Some of the patients prefer non-pharmacological treatments such as physical therapy (PT) to relieve the pain, as recent studies showed that PT may have benefits for pain management and improvement of quality of life in patients with LBP^4^. The use of surgical intervention to relieve back pain is a subject of ongoing debate, with many techniques currently being developed. However, there are still unanswered questions regarding its effectiveness^3,5^. Despite having access to conventional treatments, some patients increasingly turn to complementary and alternative medicine and non-pharmacological treatments to alleviate back pain and discomfort^6^. According to recent studies, available non-pharmacological therapies for LBP include exercise, spinal manipulation, massage (such as osteopathy), cognitive behavioural and operant therapy, acupuncture, PT (such as superficial heat or cold, ultrasound, and transcutaneous electrical nerve stimulation), bloodletting, and multidisciplinary rehabilitation^7,8^. The effectiveness of physical therapy is a subject of controversy due to the costs involved, the time it takes, and the response rate. However, it is important to consider all factors when determining its cost-effectiveness^4,8^.

In the literature review, some studies recommend acupuncture for musculoskeletal conditions such as ankle sprain, tendonitis, frozen shoulder, lumbar disc herniation, and pain management and disability improvement amongst those with CLBP^5,9^. Two traditional medicine systems widely practiced are Traditional Chinese Medicine (TCM) and Traditional Persian Medicine (TPM)^10^. Acupuncture is one of the main treatment modalities for musculoskeletal pain in TCM. Various clinical trials and systematic reviews have evaluated the therapeutic effects of acupuncture on back pain, including analgesic and muscle-relaxing effects^5,11^. Bloodletting techniques, such as cupping and venesection, have been used centuries to relieve musculoskeletal pain. While previous studies reported significant effectiveness of venesection in the treatment of different types of disease such as epistaxis, hepatitis, migraine, bronchitis, amenorrhoea, haemorrhoids, and musculoskeletal pains^12^, several clinical trials reported the role of venesection in pain relief, rehabilitation, and improvement of the quality of life of LBP patients^13^.

The WHO guideline on CLBP admits that there is no certain treatment to alleviate it, and available treatments seem to have side effects in addition to not being proven effective. On the other hand, alternative medical interventions lack sufficient evidence to support the efficacy and safety of these modalities and compare their outcomes. Hence, in this randomized clinical trial, we aimed to compare the efficacy and the safety of acupuncture, venesection, and PT in improving low back pain and daily life function in patients with CLBP.

## Materials and methods

### Study design and registration

The current study was a single-blinded, randomized clinical trial with balanced allocation (1:1:1). It was conducted at the Department of Physical Medicine & Rehabilitation Medicine, from May to August 2022. The Consolidated Standards of Reporting Trials (CONSORT) checklist of information for clinical trials was used to report this study^14^ (Supplementary file 1, Supplemental Digital Content 1, http://links.lww.com/MS9/A405). Ethical approval permission was received from the Tehran University of Medical Sciences (ID: IR.TUMS.VCR.REC.1399.202), and the protocol of the study was registered at the Iranian Registry of Clinical Trials (IRCT) (identifier: IRCT20200425047197N1).

### Participants

All the patients with a history of long-standing idiopathic chronic low back pain (LBP>3 months) who were visiting the hospital day clinic were enroled primarily. The study’s purpose was explained to the volunteers who suffered from CLBP. Those who agreed to participate in the study were evaluated by the primary investigator for further medical investigations. None of the groups received oral analgesics or other pain medications during the study period to control the confounding biases.

The 30–70-year-old men or women presented with a history of CLBP without any underlying pathology such as inflammatory, neurogenic, discogenic, osteogenic, or other detectable spinal diseases were included in the study.

Pregnancy or lactation, prior spinal invasive intervention, history of spinal trauma, long-term treatment with corticosteroids (> 3 months), any neurological deficit, psychiatric disorders, uncontrolled diabetes or high blood pressure, uncontrolled cardiovascular disease or comorbidities, neuropathies, inflammatory or known spinal disease, substance abuse or regular use of sedative-hypnotic medications, alcohol use disorder, those on anticoagulants or anti-platelets, history of faints, syncope, vasovagal syndromes, or prior history of adverse events after bloodletting including syncope and vasovagal syndrome were considered as exclusion criteria.

### Randomization and blinding

To randomize the participants, we used the block randomization method (1:1:1). We designed six blocks (ABC, ACB, BCA, BAC, CBA, CAB) and assigned each block a number from 1 to 6, respectively. To reach the sample size (105 patients), we needed 35 blocks, so we generated 30 random numbers from 1 to 6 continuously and put them in order using an online random number generator (www.calculator.net). By meeting inclusion criteria, informed consent was obtained from the patient, and they were allocated to the first free place and entered into either the physical therapy group (PTG), acupuncture group (APG), or venesection group (VSG). According to the distinct method of the interventions, the blindness of participants was not applicable; however, outcome assessors and the data analyzer were unaware of the group’s allocation.

### Trial arms

#### Venesection group

**Figure 1:**
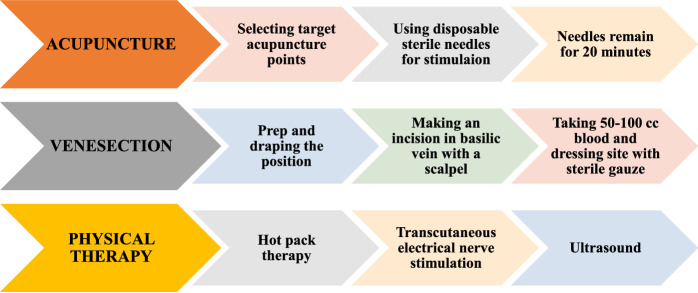
Steps of performing procedures.

**Figure 2:**
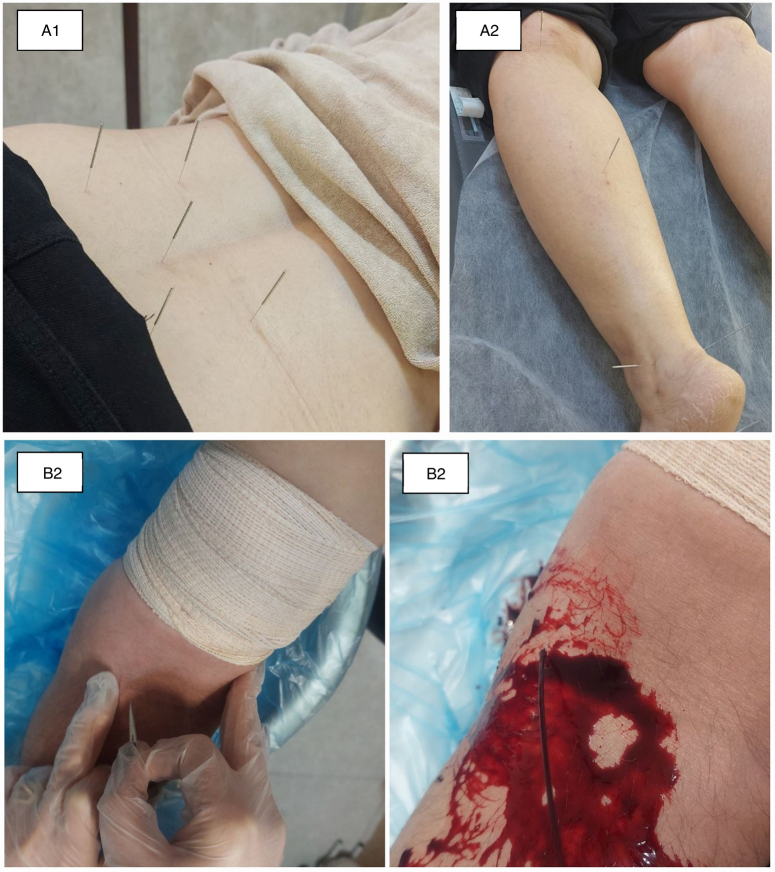
Performance of acupuncture and venesection (informed consent has been obtained from the patient). (A) Acupuncture: (A1) acupuncture points on the back and (A2) acupuncture points on the lower limb; (B) venesection: (B1) finding the basilic vein and (B2) bloodletting.

Venesection was performed two times by a TPM expert, the first session at the beginning of the study and the second session three weeks later. After prep and draping in a sterile fashion, using a number 11 sterile scalpel, 2–5 ml incisions were made in the basilic vein on the medial surface of the forearm and 50–100 ml blood was shed per session. The venesection site was dressed carefully with sterile gauze and packed. In the case of bilateral LBP, the right basilic vein was selected; however, for those with lateralized CLBP, the ipsilateral basilic vein was selected for venesection (Fig. 1 and Fig. 2A).

#### Acupuncture group

The acupuncture therapy was practiced by an experienced acupuncture specialist. The acupuncture with electrical stimulation toning of 10 Hz (SDZ-II, Hwato company, China) was performed using 0.25 × 40 mm, disposable, sterile, stainless-steel needles (Huanqiu Inc.) to a depth of 20–25 mm. Selected acupoints included: Governor Vessel-4 (GV 4) (pinyin: Ming Men, 命門), Urinary Bladder-23 (UB 23) (pinyin: Shen Shu, 腎俞), Urinary Bladder-40 (UB 40) (pinyin: Wei Zhong, 委中), Kidney-3 (Ki 3) (pinyin: Taixi, 太溪), Governor Vessel-20 (GV 20) (pinyin: Bai Hui, 百會), and the low back Ashi (local) points. The Ashi points were selected according to the acupuncturist’s examination and the patient’s condition. In each session, needles remained for 20 min and were then disposed of. The patients assigned to the APG received acupuncture therapy for their chronic low back pain three times a week for a total of 10 sessions (Figs. 1 and Fig. 2B).

#### Physical therapy group

Patients in the PT group underwent ten consecutive sessions of physical therapy three times a week, and each session lasted 1 h. Considering the treatment protocol, each session consisted of hot pack therapy (heated to 71–74°C for 20 min), transcutaneous electrical nerve stimulation (TENS; at a frequency of 100 Hz for a total of 20 min, intensity adjusted according to the patient’s tolerability; (Auvon dual channel), and ultrasound (1 MHz, 1.5 W/cm^2^ for a total of 10 min) applied on the lumbar area (Fig. 1).

### Outcome measures

Patients were evaluated three times in this study: (Time 1) at the beginning of the study (before intervention), (Time 2) 3 weeks from the start of the study that was after the last session of the intervention, and (Time 3) 1 month after the last session of the intervention. Visual analogue scale (VAS) scores were used for pain assessment, while follow-up functional outcome was assessed following the Oswestry Low Back Pain Disability Questionnaire (ODI)^15^. Adverse events during the study period were reported using direct observations or patients’ self-report statements. VAS and ODI scores were determined as the primary outcomes, while other variables were considered as secondary results. Boonstra *et al.* ^16^ reported that the reliability of VAS score for musculoskeletal pain as moderate to good, and Mousavi *et al.* ^15^ reported VAS for measuring the severity of LBP, ODI as a valid and reliable tool.

### Statistical section

#### Sample size calculation

Considering the previous studies evaluating the VAS score of pain in CLBP patients with similar study designs, considering α=0.05 and β=0.1, with the power of 90% and 15% dropout rate, the sample size was computed to be 35 patients in each arm with a total of 105 patients.

### Statistical analysis

Data were represented by mean±standard deviation (SD), frequency and percentage, mean difference (MD), and CI. Fisher exact test, analysis of variance (ANOVA), and repeated measurements were used. In the case of not-normally distributed data, appropriate non-parametric tests such as the Wilcoxon test for two related samples, Mann–Whitney U test for two independent samples, Friedman test for more than two independent samples, and Generalized Estimating Equations were used. To evaluate the effect of confounders, covariance (ANCOVA) was analyzed. An expert biostatistician performed all the analyses. *P* less than 0.05 was defined as statistical significance.

## Results

**Figure 3:**
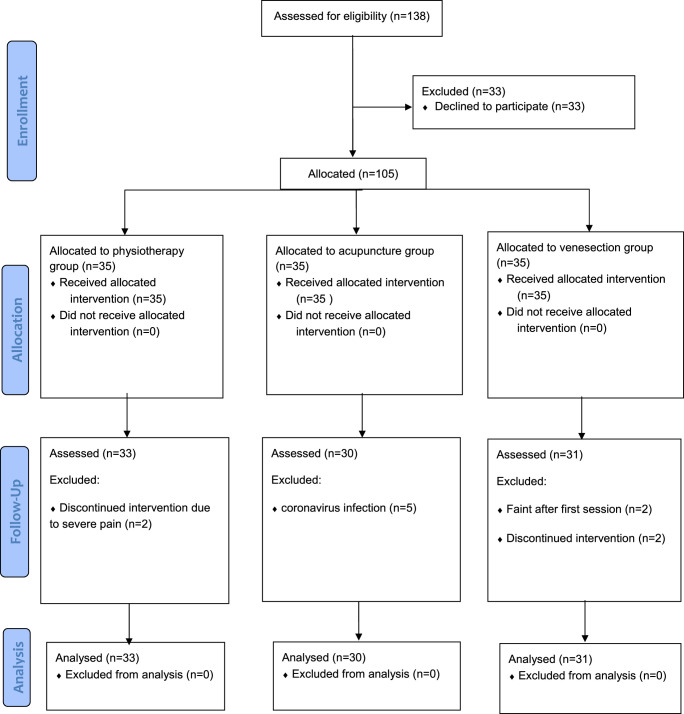
Patients’ allocation process (CONSORT flow diagram).

Ninety-four patients were reviewed for final analysis. Figure 3 represents the sequence of the study according to the CONSORT flow diagram. The mean age of patients in PTG, APG, and VSG was 46, 43, and 42 years old, respectively (Table 1, *P*=0.333). There was no significant difference between groups considering the patients' BMI (26.26 in PTG, 27.08 in APG, and 27.8 in VSG; *P*=0.309). The rest of the demographic data showed equal group randomization with no group superiority(*P*>0.05).

**Table 1:** Demographic characteristics

		Group		
	Physical therapy	Acupuncture	Venesection	*P*
Sex, *n* (%)	11 (33.3)	8 (26.7)	16 (51.6)	0.088
Male				
Female	22 (66.7)	22 (73.3)	15 (48.4)	
Age	46.39+10.70	43.47+13.80	42.239.77	0.102
BMI	26.26+2.90	27.08+4.42	27.80+4.58	0.229

There was no significant difference between the VAS scores of the patients between groups (Table 2, *P*>0.05). Immediately after the first session of each intervention, a significant reduction in VAS scores in all groups was achieved. Statistical analysis showed the mean reduction of pain severity in PTG (MD: −0.58, 95% CI: −1.43 to 0.27) was significantly lower than in VSG (MD: −3.45, 95% CI: −4.46 to −2.43) and APG (MD: −1.54, 95% CI: −2.53 to −0.54), while MD of the VSG was the highest(*P*<0.001).

**Table 2:** Comparison of LBP VAS score between groups

	VAS score (mean±standard deviation)			Mean difference (mean (95% CI))		
	Physical therapy *N*=33	Acupuncture *N*=30	Venesection *N*=31	d1 (P-A)	d2 (P-V)	d3 (A-V)
Pre	7.61±1.52	7.57±2.06	6.71±2.00	−0.04 (−0.94 to 0.86)	−0.9 (−1.78 to −0.01)	−0.86 (−1.9 to 0.18)
Post	7.03±1.94	6.03±1.77	3.26±1.98	−1 (−1.93 to −0.06)*	−3.77 (−4.74 to −2.79)***	−2.77 (−3.73 to −1.8)***
End	3.70±1.93	1.57±1.85	2.10±1.72	−2.13 (−3.08 to −1.17***	−1.6 (−2.51 to −0.68)***	0.53 (−0.38 to 1.44)
FU	5.15±1.94	1.93±2.05	2.39±1.78	−3.22 (−4.22 to −2.21)***	−2.76 (−3.69 to −1.82)***	0.46 (−0.52 to 1.44)

By performing one-way ANOVA test followed by bonferroni test as post-hoc, different letters indicate significant difference (P<0.05) and d1: Physical therapy compared to Acupuncture, d2: Physical therapy compared to Venesection, d3: Acupuncture compared to Venesection, significant level of mean difference: *P value<0.05, ***P value<0.001.

End, after the last session of treatment; FU, after 1 month follow-up; LBP, low back pain; Pre, Before treatment, Post, after the first session of treatment; VAS, visual analog scale.

During the intervention period and after the last session, the severity of LBP declined significantly in all groups. Data analysis showed that the VAS scores of VSG and APG were significantly lower than PTG (*P*<0.001). Statistical analysis showed that venesection had more dramatic pain reduction properties after the first session as compared to other modalities (*P*<0.001). The MD between VSG and APG had no remarkable difference by the end of the treatment (*P*>0.05).

**Figure 4:**
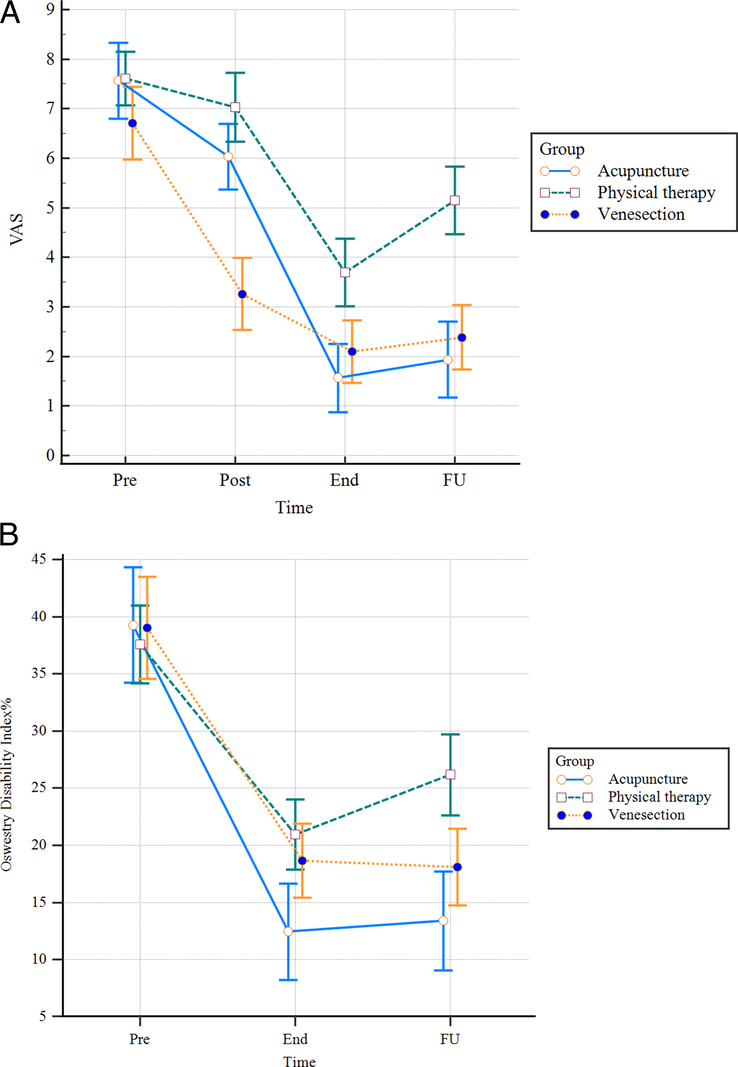
Progression of outcomes: (A) visual analogue scale (VAS) score, (B) Oswestry Disability Index (ODI)%; End, after the last session of treatment; FU, after 1-month follow-up; Post, after the first session of treatment; Pre, before treatment.

**Figure 5:**
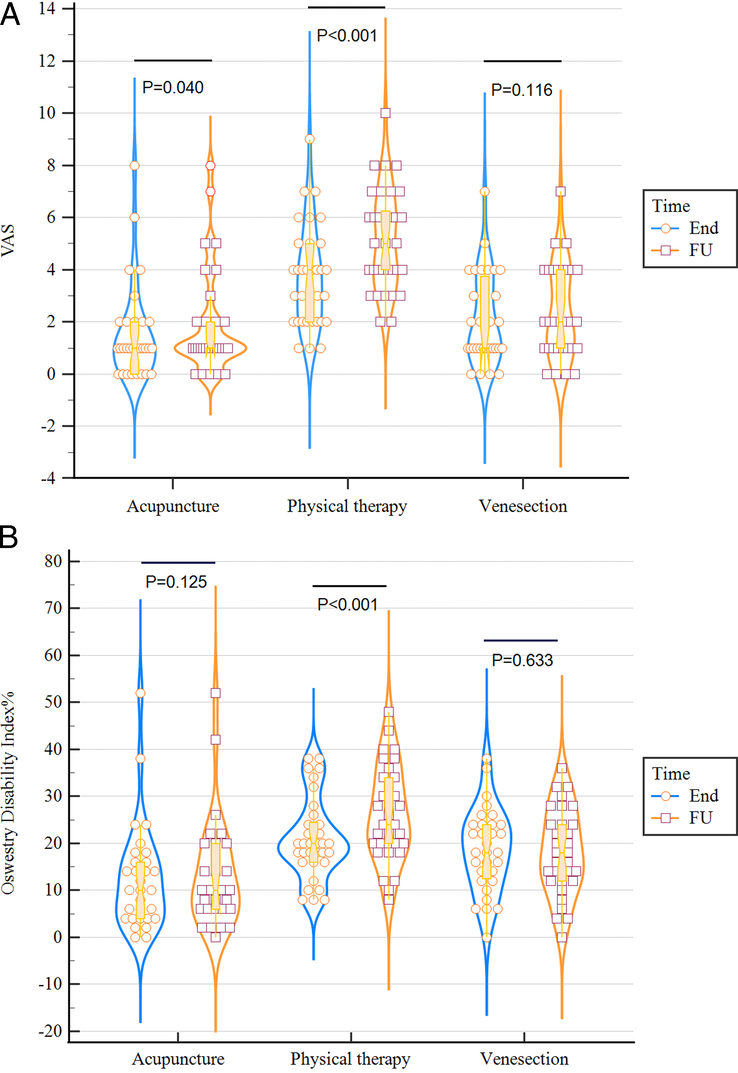
Relapse of the problems: (A) visual analogue scale (VAS) score, (B) Oswestry Disability Index (ODI)%; End, after the last session of treatment; FU, after 1-month follow-up.

Follow-up data showed that all treatment groups achieved sustainable pain reduction properties in all groups. Nevertheless, the MD of the follow-up VAS score did not differ between VSG and APG (MD: 0.46), while the mean VAS was significantly higher in PTG (*P*<0.001) (Table 2). Figure 4A shows the progression of VAS score study groups. Eventually, acupuncture and venesection both alleviated LBP more than physical therapy, even after 1 month of follow-up. Statistical analysis showed that during the follow-up period, VAS scores gradually increased in all groups; however, VSG had more sustained VAS scores at the end of treatment and during follow-up (MD: 2.9, 95% CI: −1.89 to 1.17; *P*=0.11). It is worth mentioning that in APG (MD: 0.36, 95% CI: −0.64 to 1.36; *P*=0.04) VAS was significantly lower than in other groups, and the relapse rate PTG was reported higher than the two other groups (MD: 1.45, 95% CI: 0.49–2.40; *P*<0.001) (Fig. 5A). The results of ANCOVA showed that the pain severity score at the beginning of the study significantly influenced the VAS score of the last session of intervention and follow-up; therefore, previous data analysis was performed to remove this effect.

Statistical results showed an even group distribution of the pre-operative ODI values between participants. Post-intervention ODI results showed the effectiveness of each intervention modality, regardless of the group allocation (Table 3 and Fig. 4B). The highest ODI improvement value was related to APG.

**Table 3:** Comparison of ODI between groups

	ODI score (mean±standard deviation)			Mean difference (mean (95% CI))		
Time	Physical therapy *N*=33	Acupuncture *N*=30	Venesection *N*=31	d1 (P-A)	d2 (P-V)	d3 (A-V)
Pre	37.58±9.55	39.27±13.54	39.03±12.23	1.69 (−4.17 to 7.55)	1.45 (−4.01 to 6.91)	−0.24 (−6.84 to 6.36)
End	20.97±8.62	12.47±11.27	18.65±8.81	−8.5 (−13.52 to −3.47)**	−2.32 (−6.67 to 2.03)	6.18 (1 to 11.35)*
FU	26.18±9.99	13.4±11.57	18.13±9.15	−12.78 (−18.21 to −7.34)***	−8.05 (−12.84 to −3.25)**	4.73 (−0.6 to 10.06)

By performing one-way ANOVA test followed by bonferroni test as post-hoc, different letters indicate significant difference (P<0.05); d1: Physical therapy compared to Acupuncture, d2: Physical therapy compared to Venesection, d3: Acupuncture compared to Venesection, significant level of mean difference: *P value<0.05, **P value<0.01, ***P value<0.001.

FU, after 1 month follow-up; ODI, Oswestry Disability Index; Pre, Before treatment; Post, after the first session of treatment.

Follow-up ODI in all groups was significantly lower compared to the beginning of the study; however, these results were more promising in VSG and APG. Statistical analysis showed ODI scores had a stable clinical course except in PTG, which deteriorated over the follow-up period (MD: 5.21, 95% CI: 0.62–9.79, *P*<0.001) (Fig. 5B). The results of the ANCOVA test indicated that the effect of ODI before treatment was significant; however, it was consistent among all study groups and did not impact the study’s findings.

Univariate and multivariate analysis results on the demographics (age, sex, BMI) showed no correlation or association with the primary outcomes (Supplementary file 2, Supplemental Digital Content 2, http://links.lww.com/MS9/A406).

## Discussion

CLBP is one of the leading causes of loss of work, medical expenses, and financial burdens^2^. The main purpose of LBP management is pain alleviation, improvement of spine structure impairment, and return to normal life activities as soon as possible^17^. The study compared the efficacy and safety of three different methods (physical therapy, acupuncture, and venesection) on pain severity and quality of life of patients with CLBP. The results showed that the best improvements were related to APG and VSG post-treatment and after follow-up. Recent studies have shown promising results in the treatment of CLBP with complementary therapies such as chiropractic, massage (manipulative therapy), bloodletting, cupping, herbal medicine, and acupuncture^7,18,19^. In this study, we have conducted a 3-arm randomized controlled trial (RCT) to evaluate the efficacy and the safety of acupuncture and venesection in comparison to a standard method (physical therapy). Our results were promising and confirmed that both therapies (AP and VS) significantly reduced VAS scores and improved DOI in patients with CLBP.

Many studies and several guidelines have been published to assess the effectiveness of non-pharmacological treatments, such as PT, acupuncture, or bloodletting^9,20^. A recent Cochrane review revealed that non-pharmacological methods such as acupuncture can benefit CLBP patients even immediately after the treatment^21^.

Venesection, so-called ‘fasd’ in TPM, is a particular method of bloodletting in which a small incision is made in one of the superficial veins of the body, and blood is taken. As practiced by Avicenna, this therapeutic bloodletting method is thought to alleviate pain and inflammation. From the TPM point of view, venesection is one common way for depletion. In reviewing the ancient texts of TPM, venesection is presumed to excrete portions of body waste materials. Many studies in recent years have tried to clarify the efficacy of venesection in a variety of disorders^22–24^. Previous studies showed the positive effect of venesection in the treatment of vertigo, dizziness, carpal tunnel syndrome, musculoskeletal disease, sciatica, and non-specific pains of the back, neck, and arms. The most commonly reported benefit of this technique is pain relief^12,23–25^. Amini *et al.* ^12^ hypothesized that venesection would alleviate the pain, thus improving the quality of life in patients with sciatica.

According to the literature review, venesection is considered a complementary method for the treatment of musculoskeletal or neurogenic pains^26–28^. Venesection is thought to mediate the nociceptive mechanisms of chronic pain relief^29^. Venesection alleviates pain through anti-nociceptive effects and counter-irritation, but it is still unclear how these clinical effects are generated^24,29^.

Our data revealed that venesection reduced the VAS score immediately after the first session (*P*<0.001), and its effect on pain relief was sustainable. Additionally, the patients’ functions (ODI) continued to decrease even after the last session of venesection.

Acupuncture, as a part of TCM treatment methods, is a famous non-pharmacological pain-relieving technique commonly used even in cancer-induced chronic pain^30^.

A Bayesian network meta-analysis investigated that acupuncture is the most effective and possible first-line modality for improving pain and quality of life in patients suffering from CLBP^18^. In 2016, Centers for Disease Control and Prevention (CDC) Guidelines for prescribing opioids for chronic pain and the 2017 American College of Physicians (ACP) clinical practice guidelines for management of CLBP emphasized that as a non-pharmacologic intervention, acupuncture can be included as the first-line treatment protocol for CLBP patients^31,32^.

Xiang *et al.* ^19^ conducted a systematic review with meta-analysis and found that acupuncture can provide immediate post-procedural pain reduction benefits. By contrast, a recent Cochrane review reported that acupuncture did not differ significantly from sham acupuncture in immediate pain relief after the treatment or short-term improvement of quality of life^21^.

Considering the technical points of acupuncture, the authors would like to expand the discussion in this regard. From the TCM perspective, the pathogenesis of CLBP is categorized into 3 broad types: (1) channel obstruction patterns, (2) kidney vacuity patterns, (3) or blood (or blood and qi) stasis patterns^33^. Accordingly, GV 20 raises Yang; GV 4 tonifies Kidney Qi and Yang; UB 23 Strengthens the Kidneys, tonifies Kidney Yang; UB 40 clears Heat, resolves Dampness, and activates the meridian; Ki 3 tonifies the Kidneys (Yin and Yang)^34,35^. Ashi points, also known as myofascial trigger point needling, are similar to the use of dry needling^21^.

According to previous studies BL 23 acupuncture point, facilitates the blood flow of target tissues, improves myofascial dysfunction, and promotes the recovery of damaged tissues. BL 40 may improve blood circulation and function of the nervous system by reducing myofascial tension caused by neurovascular compression. Ki 3 represents the distant point for LBP, which contains an extensive sympathetic nerve supply that plays a role in homoeostasis. GV 4 is supplied by the spinal segmental nerve and somato-somatic/visceral reflex connections with neuromodulatory effects on LBP^36,37^.

Considering the acupoint, some studies used a fixed protocol while others used a flexible set of points. Lee and colleagues and Lim and colleagues studies showed that GV 4, UB 23, UB 40, Ki 3, and local Ashi points are among the most frequently chosen choices for treating CLBP, as we used in this study^37,38^.

Classic studies suggested that the stimulation by acupuncture needles activates the inhibitory brainstem system and therefore blocks pain signals^39^. Also, many studies have demonstrated that adenosine triphosphate is triggered by an acupuncture needle. This pathway suggests a link between acupuncture and neurotransmitter signalling mediation^40,41^. Yu and colleagues demonstrated that endorphins are the most commonly reported neurotransmitter in studies that evaluated acupuncture mechanisms. They reported the effects of acupuncture in adjusting the resting state functional connectivity of major areas in the descending pain adjustment. Moreover, the amygdala can play a key role in producing anti-nociceptive effects^42^. Moreover, acupuncture reduces cyclooxygenase-2 and prostaglandin E2 levels on the peripheral level by affecting the hypothalamic-pituitary-adrenal axis, mediating peripheral opioid release^42^. Anti-nociceptive activities of acupuncture provide a better understanding of pain relief mechanisms occurring in the peripheral nervous system at a molecular level^37,43^.

In the present RCT, immediate post-procedural and post-treatment pain reduction was significantly better in APG than PTG (*P*<0.001). The authors would like to encourage further and larger RCTs to clear these controversies.

Considering adverse effects, while recent studies reported self-limited LBP, increased back pain, dizziness, or abdominal pain after PT^2^, minor haematoma, insertion point pain and swelling, nausea, minor bleeding, bruising, increased back pain, dizziness, skin reaction, and vegetative symptoms^19,21^ after receiving acupuncture, and local haematoma, dizziness, hyperventilation, anaemia, and hypotension^24,44^ In the present study only two patients fainted within 5 minutes after the first venesection session. No other adverse events were reported.

### Limitations

In the present clinical trial, we did not combine the interventions. Further studies will be required to investigate the efficacy of combining venesection with Physical therapy or complementary therapies, such as acupuncture. Although these methods improved the signs and symptoms of patients with CLBP in short-term and long-term treatments, acupuncture requires regular weekly sessions for the best outcome, and venesection is an invasive method. Because there is typically a dropout rate in studies, we calculated the dropout rate in this study. The required sample size for each group was 30 patients. We calculated 15% for dropout and collected 35 patients for each group. Because in the acupuncture group, we excluded 5 patients and 30 patients remained, we could reach the required number of patients for statistical analysis. Moreover, the sample size was not very large, which could be addressed in future research. We also suggest a longer follow-up.

## Conclusion

To our knowledge, this is the first controlled trial comparing the effectiveness of acupuncture and venesection in CLBP patients and represented the equal effectiveness of venesection and acupuncture on VAS score and ODI. This study showed that acupuncture and venesection reduced pain severity and improved the daily function of the patients with CLBP in short-term (immediately after intervention) and long-term periods (1 month after receiving the intervention). These improvements are significant compared to physical therapy, considered the standard treatment method. Thus, developing a protocol for the number of acupuncture sessions and selected acupoints seems necessary. Studies aimed at defining the number of venesection sessions and the amount of bloodletting may also be of special value. This research suggests that complementary treatments could be safe and effective for patients suffering from CLBP, as well as for short–and long-term treatment plans. These improvements are significant compared to physical therapy, considered the standard treatment method.

## Ethical approval code and trial registration

The study was supervised and approved by Tehran University of Medical Sciences ethical committee (IR.TUMS.VCR.REC.1399.202) and was registered in the Iranian registry of Clinical Trials data base (IRCT20200425047197N1).

## Consent

Written informed consent was obtained from the patient for publication and any accompanying images. A copy of the written consent is available for review by the Editor-in-Chief of this journal on request.

## Source of funding

This work was final report of the Ph.D. theses of Moein Jamali Dastjerdi and was supported by Tehran University of Medical Sciences [grant number: 46020].

## Author contribution

Conception and design: M.K., B.X.Z., and A.H.K.; acquisition and data: M.J.D., M.A., G.K., A.S., and A.H.K.; drafting the manuscript: M.S.A.M., R.A., A.S., and A.V.S.; critical revision: all authors; statistical analysis: M.S.A.M.; supervision: A.H.K. All authors approved the final version of the manuscript and agreed to be accountable for all aspects of the study.

## Conflicts of interest disclosure

None.

## Research registration unique identifying number (UIN)

This study was registered in the Iranian registry of Clinical Trials data base (IRCT20200425047197N1). https://en.irct.ir/trial/47512.

## Guarantor

Amir Hooman Kazemi.

## Data availability statement

Data can be available upon reasonable request.

## Provenance and peer review

Not commissioned, externally peer-reviewed.

## Supplementary Material
