# Supplementary material for: Comparative efficacy of acupuncture, venesection, and physical therapy on chronic low back pain outcomes: a randomized clinical trial
Source: Ann Med Surg (Lond). 2024 Mar 18;86(5):2729–38. doi: 10.1097/MS9.0000000000001944 (PMC11060228; doi:10.1097/MS9.0000000000001944)
Supplement: Supplementary file 2 [file ms9-86-2729-s002.docx]

Supplementary tables:

VAS

Supplementary table 1. ANCOVA analysis of VAS in ending time

| **Tests of Between-Subjects Effects** | | | | | |
| --- | --- | --- | --- | --- | --- |
| VAS END | | | | | |
| Source | Type III Sum of Squares | df | Mean Square | F | Sig. |
| Corrected Model | 12872.004^a^ | 6 | 2145.334 | 7.269 | .000 |
| Intercept | 1244.688 | 1 | 1244.688 | 4.217 | .043 |
| sex | 310.590 | 1 | 310.590 | 1.052 | .308 |
| age | 668.464 | 1 | 668.464 | 2.265 | .136 |
| BMI | 709.572 | 1 | 709.572 | 2.404 | .125 |
| VAS before | 2566.928 | 1 | 2566.928 | 8.697 | .004 |
| Group | 7082.107 | 2 | 3541.053 | 11.998 | .000 |
| Error | 25676.932 | 87 | 295.137 |  |  |
| Total | 96800.000 | 94 |  |  |  |
| Corrected Total | 38548.936 | 93 |  |  |  |
| a. R Squared = .334 (Adjusted R Squared = .288) | | | | | |

Supplementary table 2. ANCOVA analysis of VAS in follow up time

| **Tests of Between-Subjects Effects** | | | | | |
| --- | --- | --- | --- | --- | --- |
| VAS follow up | | | | | |
| Source | Type III Sum of Squares | df | Mean Square | F | Sig. |
| Corrected Model | 22299.584^a^ | 6 | 3716.597 | 10.473 | .000 |
| Intercept | 12.070 | 1 | 12.070 | .034 | .854 |
| sex | 4.160 | 1 | 4.160 | .012 | .914 |
| age | 494.822 | 1 | 494.822 | 1.394 | .241 |
| BMI | 49.350 | 1 | 49.350 | .139 | .710 |
| vas before | 2186.055 | 1 | 2186.055 | 6.160 | .015 |
| Group | 16841.065 | 2 | 8420.533 | 23.728 | .000 |
| Error | 30874.884 | 87 | 354.884 |  |  |
| Total | 150200.000 | 94 |  |  |  |
| Corrected Total | 53174.468 | 93 |  |  |  |
| a. R Squared = .419 (Adjusted R Squared = .379) | | | | | |

Supplementary table 3. ANCOVA analysis of ODI in ending time

| **Tests of Between-Subjects Effects** | | | | | |
| --- | --- | --- | --- | --- | --- |
| ODI END | | | | | |
| Source | Type III Sum of Squares | df | Mean Square | F | Sig. |
| Corrected Model | 2864.781^a^ | 6 | 477.463 | 6.175 | .000 |
| Intercept | 3.418 | 1 | 3.418 | .044 | .834 |
| sex | 1.681 | 1 | 1.681 | .022 | .883 |
| age | 112.599 | 1 | 112.599 | 1.456 | .231 |
| BMI | 4.977 | 1 | 4.977 | .064 | .800 |
| ODI before | 1421.940 | 1 | 1421.940 | 18.391 | .000 |
| Group | 1261.686 | 2 | 630.843 | 8.159 | .001 |
| Error | 6726.708 | 87 | 77.318 |  |  |
| Total | 38344.000 | 94 |  |  |  |
| Corrected Total | 9591.489 | 93 |  |  |  |
| a. R Squared = .299 (Adjusted R Squared = .250) | | | | | |

Supplementary table 4. ANCOVA analysis of ODI in follow up time

| **Tests of Between-Subjects Effects** | | | | | |
| --- | --- | --- | --- | --- | --- |
| ODI follow up | | | | | |
| Source | Type III Sum of Squares | df | Mean Square | F | Sig. |
| Corrected Model | 4274.520^a^ | 6 | 712.420 | 7.786 | .000 |
| Intercept | 36.745 | 1 | 36.745 | .402 | .528 |
| sex | 91.121 | 1 | 91.121 | .996 | .321 |
| age | 227.833 | 1 | 227.833 | 2.490 | .118 |
| BMI | 9.414 | 1 | 9.414 | .103 | .749 |
| ODI before | 1368.566 | 1 | 1368.566 | 14.957 | .000 |
| Group | 2568.959 | 2 | 1284.480 | 14.038 | .000 |
| Error | 7960.714 | 87 | 91.502 |  |  |
| Total | 47784.000 | 94 |  |  |  |
| Corrected Total | 12235.234 | 93 |  |  |  |
| a. R Squared = .349 (Adjusted R Squared = .304) | | | | | |
